# Blood pressure in dementia, mild cognitive impairment, and subjective cognitive decline related to time of death

**DOI:** 10.1002/brb3.2166

**Published:** 2021-05-09

**Authors:** Knut Asbjørn Hestad, Knut Engedal, Geir Selbæk, Bjørn Heine Strand

**Affiliations:** ^1^ Department of Health‐ and Nursing Science Faculty of Health and Social Sciences Inland Norway University of Applied Sciences Elverum Norway; ^2^ Department of Research Innlandet Hospital Trust Ottestad Norway; ^3^ Norwegian National Advisory Unit on Ageing and Health Vestfold County Hospital Trust Tønsberg Norway; ^4^ Department of Geriatric Medicine Oslo University Hospital Oslo Norway; ^5^ Faculty of Medicine University of Oslo Oslo Norway; ^6^ Norwegian Institute of Public Health Oslo Norway

**Keywords:** aging, blood pressure, death, dementia, mild cognitive impairment, sex, sex differences, systolic

## Abstract

**Objective:**

It is unknown whether systolic blood pressure (SBP) drop is part of the normal aging process or due to the onset of dementia for some people. SBP drop is referring to the decrease in blood pressure often seen before death. Thus, the aim of this study was to examine whether SBP at time of diagnosis of dementia, mild cognitive impairment, or subjective cognitive decline was associated with years prior to death, and whether these associations were modified by diagnoses, age, and sex.

**Methods:**

Participants were 2,236 patients from the Norwegian Registry of Persons Assessed for Cognitive Symptoms (NorCog), who died during follow‐up (2009–2017) for whom we had valid blood pressure measurements. Mean age at diagnosis was 77.5 years (*SD* 8.3), and patients were followed for an average of 3.9 years (*SD* 2.2, maximum 10.5 years). The patients had subjective cognitive decline (95), mild cognitive impairment (573), dementia (1,401), or no diagnoses related to cognitive deficits (167). SBP as dependent variable was regressed against years prior to death.

**Results:**

In men, SBP was 1.8 mmHg lower per year closer to death (*p* < .01), and this trend was linear without any acceleration. This association between years prior to death and SBP in men was not modified by age, year of diagnosis, or diagnosis. There was no such association in women.

**Conclusion:**

SBP was significantly lower for those diagnosed close to death in men, but not in women. This association was not modified by either age or onset of diagnosis. Thus, the lowering of SBP is more related to closeness to death and sex than to dementia or age. The downward trend was linear all 10 years prior to death, with no acceleration closer to death.

## Graphical abstract

For each year closer to death, the systolic blood pressure (SBP) was 1.0 mmHg lower, and the lowering of SBP was linear without any acceleration at the final years alive. However, the linear lowering was significant only in men (*p* < .01). Type of diagnosis or age was not related to the lowering of SBP.

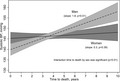

## 1 Significant Outcomes and Limitations

1. For each year closer to death, the systolic blood pressure (SBP) was 1.0 mmHg lower, and the lowering of SBP was linear without any acceleration at the final years alive.
2. The linear lowering was significant only in men (*p* < .01).
3. Type of diagnosis or age was not related to the lowering of SBP.
4. A limitation to the study is that blood pressure was only measured once, at the time of diagnosis. No longitudinal data regarding blood pressure was available.

## 2 INTRODUCTION

It is known that blood pressure drops as people approach death (Delgado et al., 2018; Goodwin, 2018; Hestad et al., 2020). Delgado et al., among others, have demonstrated in people over the age of 60 that blood pressure begins a downward trajectory more than 10 years before death, with the steepest declines occurring 2 years prior to death (Delgado et al., 2018). On the other hand, studies including people with hypertension and vascular disorders have revealed that hypertension may result in greater risk of cardiovascular disorders and death (Banegas et al., 2018; Yang et al., 2019), and it is well known that systolic hypertension in midlife increases the risk of vascular dementia (VaD) and dementia due to Alzheimer’s disease (AD) (Joas et al., 2012; Kilander et al., 1998; Kimm et al., 2011; Kivipelto et al., 2001; Launer et al., 1995; Livingston et al., 2017; Swan et al., 1998; Swan et al., 1998; Whitmer et al., 2005; Yamada et al., 2003). Lv et al., found an a U‐shape curve in systolic blood pressure (SBP) related to all‐cause mortality in a study of old Chinese people, with a mean age of 92.1 years (Lv et al., 2018). Minimum mortality risk was reported to SBP at 129 mmHg. Participants with an SBP lower than 107 mmHg or higher than 154 mmHg had a significant greater risk of death within 3 years. In another population‐based study, including participants 80 years and older, who were on anti‐hypertensive treatment it was found that SBP <135 mmHg and >145 was associated with increased mortality (Delgado et al., 2017). In a large study of frail primary‐care patients aged 75 years and above, no increased risk of mortality risk was found in people with hypertension (Masoli et al., 2020). In that study, blood pressure <130/80 was associated with higher mortality rates. The authors concluded that aggressive BP treatment in frail older people requires further evaluation. In another population‐based study from England, mortality increased with frailty and was greatest at SBP <110 mmHg (Ravindrarajah et al., 2017). The odds of having a lower SBP than 120 were higher in the last three months before death compared with 5 years before in both treated and untreated (blood pressure) participants. The study concluded that the association of low SBP with higher mortality may be due to reverse causation, for example, that severe sickness before death may lowering blood pressure. However, a study by Williamson et al. showed that lowering the SBP to <120 mmHg (treatment) may led to a significant lower rates in all‐cause mortality in people ≥75 years of age compared to people without treatment (Williamson et al., 2016).

Blood pressure is a complex process involving the central nervous system, the autonomous system with barro receptors, hormones, and other vascular parts of the body. The Renin‐angiotensin‐aldosterone hormone system, which involve many parts of the body’s internal system is of special importance. There may be many reasons for the decline in blood pressure before death, both changes within and outside the brain. Cancer and other diseases can as well result in reduced blood pressure.

The association between dementia and high SBP in midlife is reported to diminish prior to, or close to the time when a diagnosis of dementia is made (Joas et al., 2012). As early as 1996, Skoog et al. found that SBP declined after the diagnosis of both AD and VaD (Skoog et al., 1996), and it was suggested that this drop in SBP could be a cause or consequence of the brain disease causing dementia. Zlokovic et al., has proposed a “two‐hit vascular hypothesis,” for the relationship between cerebral vascular damages and Alzheimer’s disease (Zlokovic, 2011). SBP may first create cerebral stokes, which secondly may result in Alzheimer’s deposits (Zlokovic, 2011). As a result of the cerebral damages, a reduction in blood pressure may occur. However, this is a very complex process, and is not fully understood. In the Kungsholmen project, for instance, which examined people 75 years and older, a reduction in SBP with at least 10 mmhg during three years of follow‐up was associated with cognitive decline, independent of the use of antihypertensive medication (Zhu et al., 1998). For people with dementia above 80 years of age, there seems to be a paradoxical effect, as higher SBP has been associated with better cognitive performance (Euser et al., 2009; Gabin et al., 2017; Hestad & Engedal, 2006; Hestad et al., 2005; Verghese et al., 2003). In another study, Ruitenberg et al., found that there may be an inverse association between BP and dementia risk in older persons who use antihypertensive medication (Ruitenberg et al., 2001). The risk of dementia decreased with increasing blood pressure level in that study; however, the relationship was confined to subjects on antihypertensive medication. Nevertheless, it has been suggested that excessive lowering of SBP may be harmful to older patients with cognitive impairment (Mossello et al., 2015). It is also suggested that a decline in diastolic blood pressure occurs at an earlier age than SBP in patients with severe cognitive deficits (Hestad et al., 2020).

Studies indicate that the relationship between cognitive impairment and either high or low blood pressure (BP) are more clearly found in men than in women, even though women have the largest change in blood pressure during aging (Barrett‐Connor, 2013; Hestad, Engedal, Schirmer, et al., 2020; Joyner et al., 2016).

During the aging process, an increasing number of people develop dementia, and there seems to be a close connection between vascular events and increased risk of cognitive impairment and dementia; both of vascular dementia and Alzheimer’s dementia (Zlokovic, 2011). It is also reported higher mortality rates during the progression of dementia in men compared with women. Women live longer than men, especially in the severe stages of dementia (Rizzuto et al., 2012). Thus, we questioned how blood pressure could be associated with death in patients with various degree of cognitive impairment at different sex, age, and anti‐hypertensive medication. Although, this is an observational study and not a randomized controlled study it may shed some light on the relationship between blood pressure and related to time of death among people with cognitive impairment of various degree.

Given this background, we wanted to examine how blood pressure is related to closeness of death in people who were diagnosed with dementia, mild cognitive impairment, or subjective cognitive decline (SCD). We were further interested to explore if sex differences existed, as our hypothesis was that an association between low BP and time of death exists in men, but not in women. Likewise, we were interested in examining if anti‐hypertensive medication made a difference.

## 3 METHODS

### 3.1 Data

Data were retrieved from the NorCog registry—a national research and quality registry for persons referred to the specialist healthcare service for assessment of memory impairment and possible dementia. The register is based on patient consent and has existed since 2009. Currently, 42 outpatient clinics collect data for the registry on regular patients visiting the clinics. Patients’ BPs were measured during the NorCog check‐up. Vital statistics were linked to study participants who were using the National population registry, and their unique personal identification number and time from BP measurement to date to death were recorded.

All patients included in the registry are subjected to a standardized and comprehensive clinical assessment, including a health history from the patient and an informant, cognitive testing with a neuropsychological battery covering several cognitive domains, MRI or CT of the brain, blood tests, and a neurological clinical examination. If the standard examination does not lead to a diagnosis, spinal puncture with examination of beta‐amyloid and tau‐protein is an option. For some patients, Flutemetamol‐P (amyloid PET) are also offered (https://www.helsedirektoratet.no/retningslinjer/demens; (Braekhus et al., 2011).

### 3.2 Study population (*N* = 2,236)

The eligible study population comprised people with a valid BP measurement at the NorCog check‐up at baseline and registration of death in the National Death Registry between 2009 and August 2019. Between 2009 and 2017, 6,343 patients aged 40 ± (16 patients younger than 40 years were removed) were in the registry with baseline assessment. Among these, 2,505 died before the end of follow‐up; BP had been previously measured for 2,236 of these patients. Thus, the study population consisted of 2,236 persons, of whom 1,159 were women and 1077 were men. Mean age for the study population was 77.5 years (*SD* 8.3), with a range of 45–97 years (Table 1). Information about cause of death was limited to the 1,158 patients who died before 31 December 2016 (Table 2).

**TABLE 1:** Background table: Characteristics by time to death. *N* = 2,236

	Total	Years prior to death			
		<1.5	1.5–2.9	3.0–4.9	5+
Women: *n* (%)	1,159 (100)	167 (14)	247 (21)	359 (31)	386 (33)
Men: *n* (%)	1,077 (100)	185 (17)	283 (26)	317 (29)	292 (27)
Mean age in years (*SD*)					
Women	78.3 (8.5)	81.0 (8.0)	79.7 (7.4)	78.0 (8.8)	76.5 (8.6)
Men	76.6 (8.1)	78.8 (7.2)	77.9 (8.0)	76.4 (8.0)	74.3 (8.2)
Mean SBP, mmHg (*SD*)					
Women	146.4 (23.9)	147.1 (28.5)	146.8 (24.9)	145.3 (22.6)	146.9 (22.3)
Men	146.6 (22.0)	140.5 (23.0)	145.6 (22.4)	147.2 (21.3)	151.0 (20.8)
Mean MMSE (*SD*)					
Women	21.5 (4.5)	21.4 (4.6)	21.0 (4.6)	21.4 (4.6)	21.9 (4.3)
Men	23.0 (4.7)	22.4 (4.9)	22.3 (5.0)	22.7 (4.6)	24.2 (4.1)
Diagnosis: *n* (%)					
Women^a^					
SCD	39 (100)	6 (15)	3 (8)	11 (28)	19 (49)
MCI	274 (100)	40 (15)	57 (21)	86 (31)	91 (33)
Dementia	758 (100)	107 (14)	167 (22)	235 (31)	249 (33)
Other	88 (100)	14 (16)	20 (23)	27 (31)	27 (31)
Men^b^					
SCD	56 (100)	8 (14)	12 (21)	14 (25)	22 (39)
MCI	299 (100)	56 (19)	59 (20)	89 (30)	95 (32)
Dementia	643 (100)	101 (16)	187 (29)	197 (31)	158 (25)
Other	79 (100)	20 (25)	25 (32)	17 (22)	17 (22)

^a^ 88 of the women had no cognitive diagnosis.

^b^ 79 of the men had no cognitive diagnosis.

**TABLE 2:** Cause of death (ICD‐10) by 31 December 2016. Number of deaths: 1,158

	# Deaths	%
Cardiovascular disease (I00–I99)	315	28%
Cerebrovascular diseases (I60–I69)	103	9%
Diseases of the nervous system and the sense organs (G00–H95)	208	17%
Mental and behavioral disorders (F01–F99)	154	13%
Diseases of the respiratory system (J00–J99)	85	8%
Diseases of the musculoskeletal system/connective tissue (M00–M99)	5	0%
Diseases of the digestive system, (K00–K92)	29	2%
Endocrine, nutritional and metabolic diseases (E00–E89)	27	2%
Diseases of the genitourinary system (N00–N99)	33	3%
Cancer (C00–D48)	148	13%
Other causes	170	13%
Total	1,291	100%

### 3.3 Diagnosis

All information from the comprehensive baseline diagnostic work‐up, which was used to define a diagnosis, is described in detail elsewhere (Braekhus et al., 2011). Dementia diagnoses in NorCog as cause of death (from the Norwegian Death Cause Registry) are based on the International Classification of Diseases (ICD‐10) criteria for research (WHO, 1993) and made by experienced neurologists, geriatricians, or geriatric psychiatrists. The Winblad criteria were used for Mild Cognitive impairment (MCI) (Winblad et al., 2004), and patients who complained about cognitive decline but showed no cognitive impairment were given the diagnosis of SCD (Jessen et al., 2014). Patients were diagnosed as having SCD (95), MCI (573), dementia (1,401), or had no diagnosis related to cognition (167). Although it is a discussion whether SCD is more related to depression and anxiety, or if it represents the first symptoms related to dementia, we found it useful to include people with this diagnosis (Balash et al., 2013; Jessen et al., 2014). Using the Winblad criteria for MCI, the persons with such a diagnosis should be judged as not normal, nor being demented. For some, this is a sub clinical stage of dementia, which ultimately may result in dementia, others may revert to normal functioning after some time (Winblad et al., 2004). Functional activities should mainly be preserved or minimally impaired, but there should be evidence of cognitive deficits as subjective‐report, or objectively measured decline over time. Activities of daily living should be minimally or not impaired (Winblad et al., 2004). The MCI criteria is mainly based on clinical judgement, but in the present study we used the comprehensive test battery in NorCog to support the judgement (Braekhus et al., 2011).

### 3.4 Statistical methods

The association between baseline BP, as the dependent variable and years prior to death as the independent variable was used in a linear regression. Adjustment variables were restricted to age, sex, and year of diagnosis (as well as diagnosis in a supplementary analysis) as sex and age are potential confounders, and year of diagnosis is adjusted for because those diagnosed later has shorter maximum follow‐up time. Interactions between years prior to death and the variables of age and sex were investigated. To investigate possible non‐linear relationships, we applied multivariable regression splines, where years prior to death was modeled with cubic splines (mvrs package in Stata). To investigate possible homoskedasticity and influence of outliers, residuals and studentized residuals were plotted and visually investigated in kernel density plots and in a two‐way plot with the main exposure variable (years prior to death) along the X‐axis. No severe deviation from normality or homoskedasticity was observed. A sensitivity analyses was re‐run excluding outliers (outside +‐ 2 studentized residuals, *N* = 98 (4.4%).

### 3.5 Ethics Statement

The study was approved by the Regional Ethics Committee for Medical and Biological Research (REK: 2019/316), and all participants provided written informed consent.

## 4 RESULTS

The time between BP measurement at baseline and death ranged from 7 days to 10.5 years, with an average of 3.9 years (*SD* 2.2). Women had, on average, significantly more years prior to death (4.0 years [*SD* 2.0]) than did men (3.7 years [*SD* 2.2]) (test for difference: *p* < .01). Older age was significantly associated with fewer years to death in both men and women (Table 1).

**FIGURE 1:**
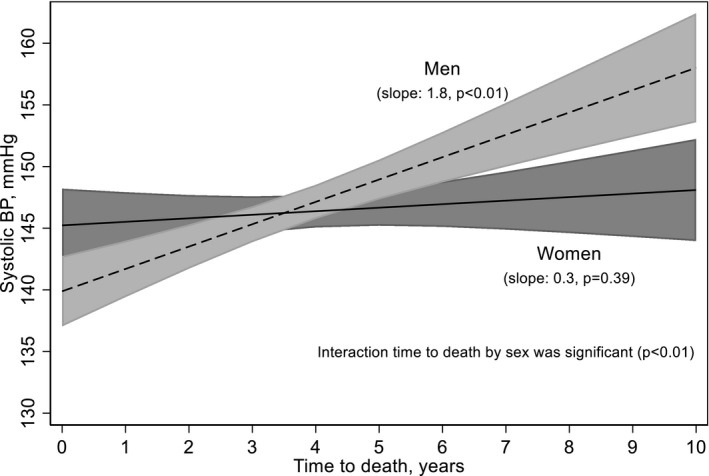
Time till death and blood pressure, adjusted by age and year of diagnosis. *N* = 2,236.

For each year closer to death, SBP was 1.03 mmHg lower (95% confidence interval (CI) 0.53, 1.54), but the association between years prior to death and SBP was significant only for men but not for women, with 1.81 mmHg lower SBP per year closer to death in men (95% CI 1.14–2.48). The corresponding number in women was 0.29 mmHg (95% CI −0.37, 0.94); Figure 1. Furthermore, a formal test of the interaction sex*years prior to death was statistically significant (*p* = .001), suggesting the association differed according to sex. The more complex model, in which years prior to death was modeled using cubic splines to capture nonlinear associations did not provide a better model fit, so this simpler linear model was preferred. Thus, there were no acceleration in the SBP drop one to two years immediate before death. Actually, the drop was equally large each year, the whole 10‐year period before death.

With blood pressure as the dependent variable, there were no significant interactions between the independent variables age and years prior to death (*p* = .3), between years prior to death and year of BP measurement (*p* = .3), or between years prior to death and cognitive diagnosis (*p* = .8). In short, the association between years prior to death did not differ across the levels of any of these three variables.

The sensitivity analysis, excluding outliers, did not impact results noteworthy and had no impact on the conclusions.

## 5 DISCUSSION

For each year closer to death, SBP was 1.03 mmHg lower, and the lowering of SBP was linear without acceleration at the final years of life. The linear lowering was significant only in men, however (*p* < .01). We cannot find any obvious explanation for this difference, although we do have some suggestions. Men and women differ in the production of various hormones over their life spans. Before menopause, women may be protected from both death and dementia by their estrogen production. Joyner et al., (2016) have suggested that high levels of estrogen may be related to physiological control of BP (Joyner et al., 2016). But their finding cannot be responsible for the observed sex differences of SBP in late life after menopause with reduced estrogen, as we found a much higher SBP in men than in women five years before death. Since we only had one measurement on each participant, it could be that situational factors like anxiety, stress, and state‐based factors influenced the blood pressure measurement. But it is no reason to believe that this should be different in men and women. It is more likely that the tendency for decline in SBP closer to death in men can be related to differences in cardio‐vascular differences between the sexes. It has for a long time been known that men have a lower survival rate than females after a diagnosis of dementia (Rizzuto et al., 2012) and there are differences in blood pressure development, cardio vascular disease, and myocardial incident of infarction between the sexes as they age (Albrektsen et al., 2016; Barrett‐Connor, 2013; Hestad, Engedal, Schirmer, et al., 2020). It is seen that there is an accelerated risk of myocardial infarction in young men compared to young women. It decreases with aging (Hestad et al., 2020), but this gap, however, never disappears even if it becomes smaller (Hestad et al., 2020; Barrett‐Connor, 2013). There is also a tendency for women to experience cardiovascular disease 6–10 years later than men. Our finding related to SBP and death may reflect this delay in women, with no indication of relationship between SBP decline and death.

Different incidences of myocardial infarction (MI) for men and women, at least until age 95, could be related to differences in sympathetic nerve activity between old men and old women (Barrett‐Connor, 2013; Daka et al., 2015). Daka et al., (2015) have suggested that the lower incidence rates of MI in women may be caused by different endothelin‐1 levels. Both suggestions—the difference in sympathetic activity and in endothelin‐1 level between old men and women—could influence the incidence rate of MI and death. Our data show that a large number of persons in our study died of cardiovascular disease.

However, to be sure that state‐based factors rather than long‐term cerebrovascular health is the important mediator of differences in SBP related to death, longitudinal studies are required.

Another possible explanation for the sex difference found in this study may be related to differences in cell senescence pathways, which, in turn, may contribute to increased longevity in women and may also limit organ damage caused by hypertension (Colafella & Denton, 2018). This explanation is unlikely, however, as age did not influence the association between SBP and years prior to death.

We hypothesized that cognitive diagnosis would play a role related to time of death, but it did not. Neither did degree of cognitive performance as measured by the MMSE influence number of years prior to death. It could be the MMSE variation in test performance among participants was too small for a prediction in a statistical model. The MMSE scores were around 21–24 and the SDs were about 4–5. It is reasonable to believe, however, that this fall in BP is part of a terminal decline, possibly related to cardiovascular disease (Delgado et al., 2018) and not specifically related to cognitive deficits of minor or major degree.

Our main intention with the present study was to investigate whether we could replicate the findings of a drop in blood pressure as people approach death, and to expand on these findings to investigate whether this trend differed according to the diagnosis of dementia, MCI, or SCI. Further, as this estimate is per year, in men, it means that 5 years prior to death the systolic BP is 9.0 mmHg larger than immediately before death, which is indeed a substantial and meaningful effect size.

Our data clearly show that there are differences regarding blood pressure and death in the process of aging between men and women. In the present study particularly related to survival after a diagnosis of dementia, MCI, or SCI. For the clinician, it can be difficult to know if the low blood pressure is related to a cognitive disorder or if it could be an indication of general deteriorating of health, especially in men. Underlying poor cardio‐vascular health in people with dementia should be screened for and treated if indicated.

The strength of this study is the large number of patients in our sample, although it does have some obvious limitations, including lack of a control group from a healthy population. The study was cross‐sectional, in which patients’ BPs were studied at one point, with no longitudinal follow‐up data of SBP. Although health personnel working at the various centers were instructed in performing the standardized assessment, including the procedure for BP measurement, we cannot be positive that all patients were examined in exactly the same way. Furthermore, we do not know about the patients’ BP history before diagnosis.

## 6 CONCLUSION

Systolic blood pressure decreases significantly from time of diagnosis of cognitive decline to death in men, but not in women. The SBP drop was not modified by age or by diagnosis of cognitive deficits.

## CONFLICT OF INTEREST

The authors have nothing to disclose. The authors declare that the research was conducted in the absence of any commercial or financial relationships that could be construed as a potential conflict of interest.

### ETHICAL APPROVAL AND INFORMED CONSENT

The study was approved by the Regional Ethics Committee for Medical and Biological Research (REK: 2019/316), and all participants provided written informed consent.

## AUTHOR CONTRIBUTIONS

BS did the statistical analyses. KH wrote the first draft of the manuscript. BS and GS collected the data. All authors took part in the writing process, initiation, and planning of the study.

### PEER REVIEW

The peer review history for this article is available at https://publons.com/publon/10.1002/brb3.2166.

## Data Availability

The data analyzed in this study was subjected to the following licenses/restrictions: The data were collected from the Norwegian Register of Persons Assessed for Cognitive Symptoms (NorCog). The data can be available after approvement from the board of the database. Requests to access these datasets should be directed to Marit Nåvik, naam@sthf.no.
